# MDWC-Net: a multi-scale dynamic-weighting context network for precise spinal X-ray segmentation

**DOI:** 10.3389/fphys.2025.1651296

**Published:** 2025-08-29

**Authors:** Zhongzheng Gu, Xuan Wang, Baojun Chen

**Affiliations:** ^1^ Department of Spine and Spinal Cord Surgery, Henan Provincial People’s Hospital, Zhengzhou, Henan, China; ^2^ Department of Medical Imaging, The Third Affiliated Hospital of Zhengzhou University, Zhengzhou, Henan, China

**Keywords:** convolutional neural networks, spinal image segmentation, multi-scale convolutional adaptive weighting, dual feature complementary block, bottleneck information enhancement block

## Abstract

**Purpose:**

Spinal X-ray image segmentation faces several challenges, such as complex anatomical structures, large variations in scale, and blurry or low-contrast boundaries between vertebrae and surrounding tissues. These factors make it difficult for traditional models to achieve accurate and robust segmentation. To address these issues, this study proposes MDWC-Net, a novel deep learning framework designed to improve the accuracy and efficiency of spinal structure identification in clinical settings.

**Methods:**

MDWC-Net adopts an encoder–decoder architecture and introduces three modules—MSCAW, DFCB, and BIEB—to address key challenges in spinal X-ray image segmentation. The network is trained and evaluated on the Spine Dataset, which contains 280 X-ray images provided by Henan Provincial People’s Hospital and is randomly divided into training, validation, and test sets with a 7:1:2 ratio. In addition, to evaluate the model’s generalizability, further validation was conducted on the Chest X-ray dataset for lung field segmentation and the ISIC2016 dataset for melanoma boundary delineation.

**Results:**

MDWC-Net outperformed other mainstream models overall. On the Spine Dataset, it achieved a Dice score of 89.86% ± 0.356, MIoU of 90.53% ± 0.315, GPA of 96.82% ± 0.289, and Sensitivity of 96.77% ± 0.212. A series of ablation experiments further confirmed the effectiveness of the MSCAW, DFCB, and BIEB modules.

**Conclusion:**

MDWC-Net delivers accurate and efficient segmentation of spinal structures, showing strong potential for integration into clinical workflows. Its high performance and generalizability suggest broad applicability to other medical image segmentation tasks.

## 1 Introduction

The spine, as the structural support for the body and its organs, can develop deformities, cause back pain, or even lead to paralysis when affected by disease (Khalifeh et al., 2024). Accurate spinal segmentation plays a crucial role in the diagnosis and treatment of spinal disorders. It not only enables clinicians to more precisely locate and identify spinal structures, but also provides the foundation for measuring key spinal parameters, spinal registration, and scoliosis classification (Liebmann et al., 2024; Azampour et al., 2024; Sarwahi et al., 2021; Thibodeau-Antonacci et al., 2025; Kim et al., 2022). Such technology is essential for evaluating disease severity, monitoring progression, and planning surgical interventions. Among the various imaging modalities, X-ray technology has become a commonly used clinical tool for spinal disease diagnosis due to its advantages of low radiation exposure, rapid imaging, and cost-effectiveness (Zhang et al., 2020). Consequently, developing automatic segmentation algorithms tailored for spinal X-ray images holds substantial clinical value. In recent years, deep learning—an emerging branch of artificial intelligence—has achieved remarkable progress in image classification, semantic segmentation, and object detection, by learning high-level representations from data (Duan et al., 2025; J. Chen et al., 2025; Zhou et al., 2023; Gui et al., 2024). These advancements offer innovative solutions for accurate segmentation in spinal medical imaging. However, current deep learning-based methods for spinal X-ray image segmentation still face several limitations. First, spinal structures often exhibit complex multi-scale characteristics, and existing methods struggle to capture features at different scales effectively, resulting in suboptimal segmentation performance and loss of fine details. Additionally, many networks suffer from inadequate feature fusion mechanisms, which leads to redundancy and information loss. To address the above challenges, we propose a novel deep learning framework, the Multi-Scale Dynamic-Weighting Context Network (MDWC-Net), for spinal X-ray image segmentation. The network is designed to enhance the extraction of anatomical details across multiple scales, improve the fusion between low- and high-level features, and strengthen the modeling of global contextual information. The main contributions of this work are as follows:

1. A specialized segmentation framework tailored for spinal X-ray images is developed, aiming to provide a reliable and efficient tool to support automatic diagnosis and quantitative analysis in clinical settings.
2. Effective modules are designed to improve multi-scale representation, contextual awareness, and feature interaction within the encoder–decoder architecture.
3. Extensive experiments on spinal and cross-modality datasets demonstrate the superior performance, efficiency, and generalization ability of our proposed method.

## 2 Materials and methods

### 2.1 Application of deep learning techniques in medical image segmentation

In recent years, deep learning techniques have made significant progress in various fields. Deep learning automatically extracts features from data through multi-layer neural networks, eliminating the complex process of traditional feature engineering (Talaei Khoei et al., 2023). Medical image segmentation, as one of the key tasks in medical image processing, aims to separate the region of interest from the background in images, helping clinicians with disease diagnosis and treatment. Fully Convolutional Networks (FCNs) are among the earliest deep learning models to achieve significant progress in medical image segmentation tasks (Wang et al., 2022). By replacing the fully connected layers in traditional convolutional neural networks with convolutional layers, FCNs are capable of performing pixel-level classification on input images of any size. FCN-8s is a variant of FCN that fuses feature information from different layers to enhance segmentation accuracy. The DeepLab series is another classic segmentation network model, including DeepLabV1, DeepLabV2, and DeepLabV3 (Yang et al., 2024; Jeong et al., 2024; L. C. Chen et al., 2018). DeepLabV1 extends the receptive field of convolutions through dilated convolutions, effectively improving the segmentation capability for medical images with complex backgrounds or unclear edges. DeepLabV2 builds upon this by incorporating Conditional Random Fields (CRFs) for post-processing, refining the segmentation results. DeepLabV3 further improves the dilated convolution and combines it with an encoder-decoder architecture, enabling it to handle more complex medical image segmentation tasks.

Compared to the classic models mentioned above, U-Net, which is currently the most widely used model in the field of medical image segmentation, was first introduced in 2015 (Falk et al., 2019). It adopts an encoder-decoder architecture and combines low-level features with high-level features through skip connections, preserving the spatial information of the image. Due to its superior segmentation performance, U-Net has also been introduced into industrial fields such as defect detection (Xia et al., 2023; Tulbure et al., 2022) and remote sensing image segmentation (Bai et al., 2023; J. Li et al., 2022). With the deepening of research, more and more scholars have proposed various improvements to address the shortcomings of U-Net, resulting in numerous variant models (Das and Das, 2024; Zhou et al., 2024; Tang et al., 2024; Jisna et al., 2024; Chen et al., 2024; Li et al., 2023) for different segmentation tasks. These network models are widely applied to segmentation tasks in medical images such as those of the heart, liver, blood vessels, and cells (Carneiro et al., 2012; Khan et al., 2022; Gegundez-Arias et al., 2021; Greenwald et al., 2022; Le, 2023). Zhao et al. (2021) introduced a multi-scale up-sampling attention block to enhance feature representation and adopted a nested skip-connection pyramid architecture for feature extraction, applying it to the retinal vessel segmentation task. Li et al. (2023) integrated an attention context encoding module and dual segmentation branches, improving liver segmentation accuracy while keeping the parameter count reasonable. Zhu et al. (2023) used the Swin Transformer framework to extract semantic features and introduced a shift-block labeling strategy during training to achieve more precise brain tumor segmentation. Zhao et al. (2022) focused on two main aspects—sequence encoding and variational information bottlenecks—and proposed an improved model based on different deep learning architectures for peptide toxicity prediction. Although transformer-based models like Swin Transformer have achieved success in brain tumor and peptide segmentation, their application to spinal X-ray segmentation remains limited due to the modality’s lower contrast and structural complexity.

### 2.2 Application of deep learning techniques in spinal image segmentation tasks

The segmentation of spinal images aims to assist doctors in better understanding the patient’s condition. H. Li et al. (2021) improved model accuracy by embedding a dual-branch multi-scale attention module. This method achieves the segmentation of vertebrae, laminae, and the dural sac from lumbar MRI images, thereby providing assistance in diagnosing lumbar spinal stenosis. Shi et al. (2022) designed a novel dual-path network based on an attention gate (AGNet). This model consists of a context path and an edge path, aiming to extract semantic and boundary information from the spinal and vertebral regions. A multi-scale supervision mechanism is employed to explore comprehensive features, and an edge-aware fusion mechanism is used to combine the features extracted from both paths, enhancing segmentation performance. Chen et al. (2024) combined U-Net and Mask R-CNN to achieve automatic segmentation and labeling of vertebrae in lateral cervical and lumbar X-ray images, with accuracy improved through rule-based strategies. Deng et al. (2024) proposed a complementary network that integrates the advantages of U-Net and BiseNet for spinal segmentation in MRI images. The network uses strip pooling (SP) blocks to replace the spatial extraction path in the BiseNet framework and employs an attention refinement module to fuse the extracted features, thereby improving segmentation accuracy. While multi-scale and attention-based methods have shown success in MRI and CT segmentation, their direct application to spinal X-rays is limited due to lower soft-tissue contrast, overlapping anatomical structures, and higher noise. Although a few studies have begun exploring such strategies in X-ray contexts, their effectiveness remains constrained. To address the challenges in spinal X-ray image segmentation, we propose MDWC-Net, which integrates task-specific feature weighting and structure-aware fusion strategies. The model enhances both local detail capture and global contextual understanding.

### 2.3 Overall network architecture

The task of spinal image segmentation often faces numerous challenges, including the complexity of spinal structures, noise interference in images, and the inability to effectively fuse features of different scales. Due to the diverse presentation of the spine in X-ray images and the presence of similar backgrounds, the segmentation process struggles to accurately capture details and boundaries. Furthermore, the lack of effective utilization of features at different scales can lead to a decrease in segmentation accuracy.

**FIGURE 1:**
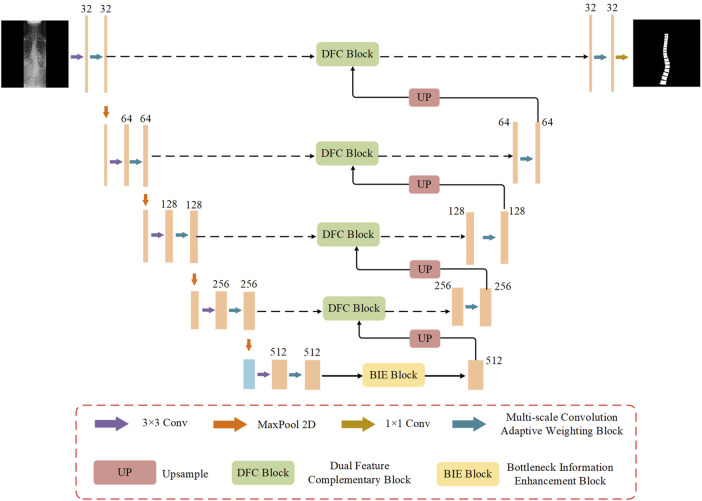
The overall network structure of MDWC-Net.

To address these issues, Multi-Scale Dynamic-Weighting Context Network (MDWC-Net) is proposed. As shown in Figure 1, MDWC-Net mainly consists of four parts: the encoder structure, decoder structure, skip connections, and bottleneck structure. MDWC-Net utilizes a multi-scale convolutional adaptive weighting block to perform feature extraction and target reconstruction. By jointly learning different channels of multi-scale feature maps, it dynamically adjusts the importance of different regions. The developed dual feature complementarity block enables effective fusion of high-level semantic information from the encoder structure and low-level spatial information from the decoder structure, enhancing the network’s ability to capture spinal detail information. Furthermore, a bottleneck information enhancement block is designed at the bottleneck layer of the network, allowing the network to more fully capture and utilize global contextual information, thereby strengthening the representation of key information.

### 2.4 Multi-scale convolution adaptive weighting block

**FIGURE 2:**
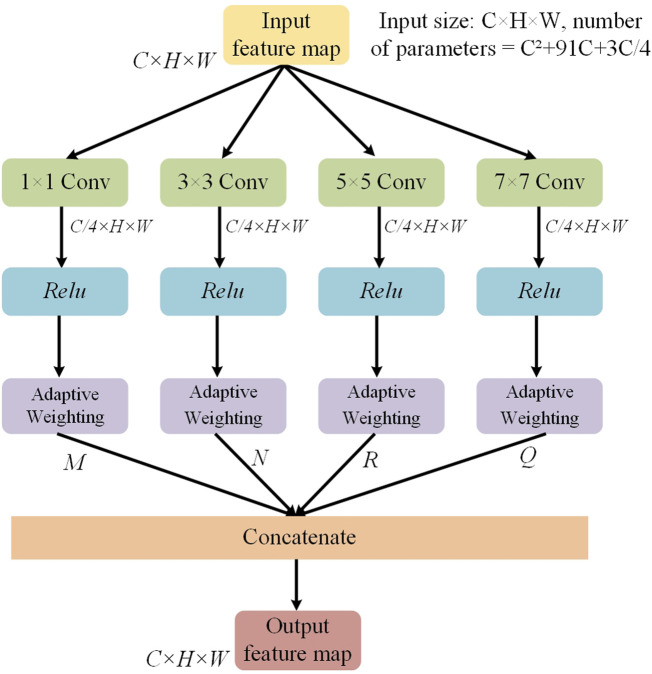
The structure of the multi scale convolution adaptive weighting block.

Figure 2 shows the structure of the multi-scale convolutional adaptive weighting block. Different scales of convolutional kernels are selected to extract multi-scale information from the input image, addressing the issue of insufficient multi-scale detail extraction capability in spinal image segmentation tasks. At the same time, an adaptive weighting block is constructed to dynamically adjust channel weights based on the feature information of different input images. Through the construction of multi-scale depthwise separable convolutions and the adaptive weighting block, the model is enabled to efficiently and thoroughly extract multi-scale features from the image while dynamically adjusting the weights of different features based on the input. This enables more accurate segmentation of the regions of interest in the spine.

Among them, the multi-scale convolution operation is the core method for achieving multi-scale feature extraction. To capture information at different spatial scales, we designed multiple convolution kernels of different sizes (1 × 1, 3 × 3, 5 × 5, 7 × 7) and performed the computations using depthwise separable convolutions. Unlike the fully connected convolutions in traditional convolutional neural networks, depthwise separable convolutions break down the convolution operation into two steps: first, performing a channel-wise convolution on each individual channel, and then performing a pointwise convolution across channels. This strategy not only effectively reduces computational complexity but also enables the simultaneous capture of small-scale features at the fine detail level and large-scale features at the global level. It provides a rich feature foundation for subsequent feature fusion and dynamic weighting.

**FIGURE 3:**
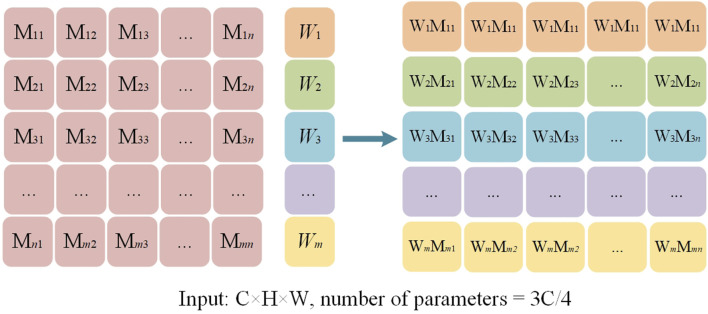
The adaptive weighted feature map.

To further optimize the utilization of features, this study designs an adaptive weighting block based on the extraction of multi-scale features. The block contains learnable weight parameters, which are optimized during the training process according to the specific feature requirements of the image. By dynamically adjusting the weights of features at different scales, MDWC-Net can more flexibly adapt to different input features, thus achieving higher accuracy in the spine region of interest segmentation task. The weighting process is shown in Figure 3, where the feature extraction with convolution kernels of sizes 1 × 1, 3 × 3, 5 × 5, and 7 × 7 corresponds to the dynamic weighted feature vectors M, N, R, and Q, respectively. Using a parallel approach, M, N, R, and Q are merged to form the multi-scale convolution adaptive weighting block.

For the adaptive weighting layer shown in Figure 3, the weight $W_{m}$ in the parameter update is calculated as follows during the gradient computation (Equation 1):

$$\nabla_{Wn}L\left(W_{n}\right)=\frac{\partial L}{\partial W_{n}}=\frac{\partial L}{\partial Y_{n}}\frac{\partial Y_{n}}{\partial W_{n}}=\sum_{j}X_{nj}\frac{\partial L}{\partial Y_{n}}$$ (1)

Where *L* represents the loss function, and $Y_{n}$ denotes the output result after feature weighting of the *n-th* row.

The weight update can be expressed as (Equation 2):

$$W_{n}^{l+1}=W_{n}^{l}-\eta \nabla W_{n}^{l}L\left(W_{n}^{l}\right)$$ (2)

Where *l* represents the number of parameter updates, η represents the learning rate, and $W_{n}^{l}$ denotes the parameter values at the $W_{n}$ iteration.

The entire module computation process is as follows (Equations 3–6):

$$F_{1}=Relu\left[k_{1}\left(X\right)\right];F_{2}=Relu\left[k_{3}\left(X\right)\right]$$ (3)

$$F_{3}=Relu\left[k_{5}\left(X\right)\right];F_{4}=Relu\left[k_{7}\left(X\right)\right]$$ (4)

$${\tilde{F}}_{1}=\delta \left(F_{1}\right);{\tilde{F}}_{2}=\delta \left(F_{2}\right);{\tilde{F}}_{3}=\delta \left(F_{3}\right);{\tilde{F}}_{4}=\delta \left(F_{4}\right)$$ (5)

$$F_{out}=Concat\left({\tilde{F}}_{1},{\tilde{F}}_{2},{\tilde{F}}_{3},{\tilde{F}}_{4}\right)$$ (6)

Where $k_{i}\left(\cdot \right)$ represents the convolution operation with a filter size of i×i , δ represents the adaptive weighting layer, and $Concat\left(\cdot \right)$ represents the concatenation along the channel dimension.

### 2.5 Dual feature complementary block

**FIGURE 4:**
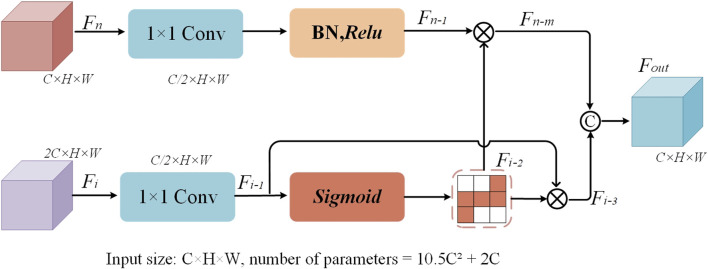
The structure of the dual-feature complementary block.

The traditional U-Net uses skip connections that directly concatenate the feature maps from the encoder and decoder parts to recover lost spatial details during the decoding process. However, this approach often leads to insufficient information fusion, especially when it comes to recovering fine details. To address this issue, an innovative dual feature complementary block has been designed, which optimizes the traditional skip connection method through a series of processing steps. This enables the network to more effectively complement and fuse the feature maps from the encoder and decoder. The structure of the dual feature complementary block is shown in Figure 4.

The core idea of the dual feature complementary block is to enhance the interaction and information transfer between feature maps by progressively optimizing the feature map fusion process. Specifically, the dual feature complementary block independently processes each feature map from the skip connections, including operations such as convolution for dimensionality reduction, batch normalization, and nonlinear activation. Then, pixel-wise multiplication is applied to enhance the mutual influence between feature maps. Finally, the results of both feature maps are fused and concatenated to form a more refined feature map representation. When processing the encoder feature map, the first step is to reduce the number of channels of feature map $F_{n}$ from C to C//2 using a 1 × 1 convolution. Next, batch normalization is introduced to stabilize the training process, preventing issues like gradient vanishing or explosion. The *Relu* activation function is then applied to introduce nonlinearity, enhancing the model’s expressive power, resulting in the feature map $F_{n-1}$ . The specific computation process is as follows (Equation 7):

$$F_{n-1}=\text{Re}lu\left[BN\left(Conv_{1\times 1}\left(F_{1}\right)\right)\right]$$ (7)

Where M, N, and P represent the 1 × 1 convolution operation, the *Relu* activation function, and the exponential function, respectively.

When processing the decoder feature map $F_{i}$ , the operation is similar to that of the encoder feature map. The feature map is first reduced in dimensionality using a 1 × 1 convolution, decreasing the number of channels from 2C to C//2. It is worth noting that in the processing of the decoder feature map, the Sigmoid activation function is used instead of *Relu*. This design choice allows for smoother interaction between feature maps, avoiding information distortion caused by excessive activation. This is particularly effective in recovering fine details. The specific computation process is as follows (Equations 8 and 9):

$$F_{i-1}=Conv_{1\times 1}\left(F_{i}\right)$$ (8)

$$F_{i-2}=Softmax\left(F_{i-1}\right)=\frac{\exp\left(F_{i-1}\right)}{\sum_{m=1}^{c}\exp\left(F_{i-1}\right)}$$ (9)

Where Conv and $\exp\left(\cdot \right)$ represent the 1 × 1 convolution operation and the exponential function, respectively, while m represents the index of each channel in the feature map $F_{i-1}$ .

After independently processing the feature maps, the dual feature complementary block performs a pixel-wise multiplication operation between the processed encoder feature map $F_{n-1}$ and the decoder feature map $F_{i-2}$ , allowing for tighter integration of detail recovery and contextual fusion, resulting in feature map $F_{n-m}$ . At the same time, to enhance the information interaction between the feature maps, a pixel-wise multiplication is performed between feature map $F_{i-1}$ and $F_{i-2}$ , resulting in feature map $F_{i-3}$ . Finally, the feature map $F_{n-m}$ and feature map $F_{i-3}$ are concatenated along the channel dimension to strengthen the network’s use of multi-level information during the skip connection phase, resulting in feature map $F_{out}$ . By organically combining these two types of feature information, the network is able to leverage both low-level and high-level features, while avoiding issues such as information loss and redundancy. The specific computation process is as follows (Equations 10–12):

$$F_{n-m}=F_{n-1}⊗F_{i-2}$$ (10)

$$F_{i-3}=F_{i-1}⊗F_{i-2}$$ (11)

$$F_{out}=Concat\left[F_{n-m};F_{i-3}\right]$$ (12)

Where ⊗ and $Concat\left(\cdot \right)$ represent the pixel-wise multiplication and concatenation operations, respectively.

### 2.6 Bottleneck information enhancement block

**FIGURE 5:**
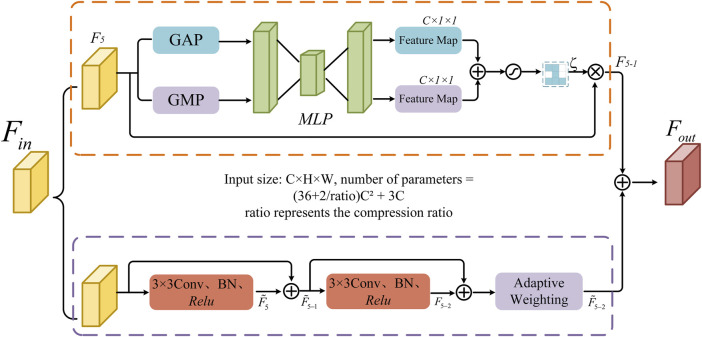
The structure of the bottleneck information enhancement block.

Convolutional neural networks often encounter issues such as feature information loss and insufficient global information capture when processing high-dimensional feature maps. To address this problem, this study designs a bottleneck information enhancement block, as shown in Figure 5. This block is constructed using parallel dual branches.

The upper branch first applies global average pooling and global max pooling operations to capture global average information and extract the maximum value from each local region. These operations provide a smooth representation of the entire image’s features and help capture prominent local features. The spatial dimensions of feature map $F_{5}$ are compressed to 1 × 1, retaining the global statistical features of each channel. The resulting features are then fed into a shared multilayer perceptron (*MLP*), which compresses and expands the input feature map along the channel dimension to extract potential high-level feature representations. Next, the outputs from both average pooling and max pooling are added together, and the Sigmoid activation function is applied to generate the weight coefficients, resulting in a normalized channel weight map ζ . Finally, the original feature map $F_{5}$ is element-wise multiplied by the channel weight map ζ , achieving weighted fusion of the feature maps to enhance their effectiveness during the decoding process. The entire operation of the upper branch is as follows (Equations 13 and 14):

$$\zeta =Sigmoid\left[\gamma \left[P_{avg}\left(F_{5}\right)\right]⊕\gamma \left[P_{\max}\left(F_{5}\right)\right]\right]$$ (13)

$$F_{5-1}=\zeta ⊗F_{5}$$ (14)

Where $P_{avg}$ and $P_{\max}$ represent the global average pooling and global max pooling operations, respectively. $\gamma \left(\cdot \right)$ denotes the *MLP* operation, and ⊕ and ⊗ represent addition and element-wise multiplication, respectively.

The lower branch utilizes two consecutive 3 × 3 convolutional layers to extract feature information. The output fused after the first convolution is fed into the second convolutional layer. Through residual connections, more information is retained during feature propagation, which helps avoid information loss in deep networks and facilitates the stable transmission of information flow. Subsequently, an adaptive weighting mechanism is introduced to enhance the key information in the feature map, resulting in feature map ${\tilde{F}}_{5-2}$ . The computation process of the lower branch is as follows (Equations 15–18):

$${\tilde{F}}_{5}=\text{Re}lu\left[BN\left(Conv_{3\times 3}\left(F_{5}\right)\right)\right]$$ (15)

$${\tilde{F}}_{5-1}={\tilde{F}}_{5}+F_{5}$$ (16)

$$F_{5-2}=\text{Re}lu\left[BN\left(Conv_{3\times 3}\left({\tilde{F}}_{5-1}\right)\right)\right]$$ (17)

$${\tilde{F}}_{5-2}=\delta \left(F_{5-2}+{\tilde{F}}_{5-1}\right)$$ (18)

where $Conv_{3\times 3}\left(\cdot \right)$ and $BN\left(\cdot \right)$ represent 3 × 3 convolution operation and batch normalization respectively, and δ represents adaptive weighting layer.

Finally, the output results from the upper and lower branches are fused to obtain the final output feature map $F_{output}$ . This process integrates multi-level feature information and the weighting mechanism, effectively enhancing the network’s expressive capability (Equation 19).

$$F_{output}=F_{5-1}⊕{\tilde{F}}_{5-2}$$ (19)

Where, ⊕ represents the pixel-wise addition operation.

## 3 Experiment and results

### 3.1 Dataset

The spine X-ray dataset used in this study was provided by the Department of Spine Surgery at Henan Provincial People’s Hospital, consisting of 280 PNG-format images. Prior to the study, all images underwent de-identification processing to protect patient privacy and were named the “Spine Dataset.” The spine regions in the images were precisely annotated by two spine surgeons using the Labelme annotation tool. To ensure annotation consistency, two spine surgeons jointly defined annotation standards and performed quality cross-checks on randomly selected samples, confirming a high level of agreement. The annotated dataset was randomly divided into training, validation, and test sets in a 7:1:2 ratio. In addition, to validate the generalization ability of MDWC-Net across different segmentation tasks, experiments were conducted using the ISIC 2016 (Gutman et al., 2016) dataset for skin lesion segmentation and the Chest X-ray dataset (Jaeger et al., 2014; Candemir et al., 2014) for lung field segmentation. Both datasets were randomly split into training, validation, and test sets using a 7:1:2 ratio. All images were uniformly resized to 256 × 256 pixels according to a proportional scaling principle.

### 3.2 Experiment setup and evaluation metrics

#### 3.2.1 Experiment setup

All models in this study were implemented using the PyTorch deep learning framework and Python 3.7, with computations performed on an NVIDIA RTX 2080Ti GPU. The batch size was set to 8, and the cross-entropy loss function was adopted. Random horizontal flipping was applied as a data augmentation technique to the training dataset. The SGD optimizer was used for model training, with an initial learning rate of 0.001. The total number of training epochs was set to 100, and the learning rate was reduced by a factor of 10 every 20 epochs.

#### 3.2.2 Evaluation metrics

To better assess the network performance, this study employs four commonly used image segmentation evaluation metrics: Global Pixel Accuracy (GPA), Dice Coefficient (Dice), Mean Intersection over Union (MIoU), and Sensitivity. Global Pixel Accuracy measures the proportion of correctly classified pixels overall. The Dice Similarity Coefficient provides a comprehensive evaluation of the overlap between the segmentation results and the ground truth labels. Mean Intersection over Union considers the degree of overlap between the predicted and ground truth labels for each class, while Sensitivity reflects the model’s ability to recognize positive class regions. In addition, this study also reports the number of parameters (Params), floating-point operations (FLOPs), and Training_time for each model to evaluate their computational efficiency. The specific expressions for these metrics are as follows (Equations 20–23):

$$GlobalPixelAccuracy=\frac{TP+TN}{TP+FP+FN+TN}$$ (20)

$$MIoU=\frac{1}{m}\sum_{i=0}^{m}\frac{TP_{i}}{FN_{i}+FP_{i}+TP_{i}}$$ (21)

$$Dice=\frac{2TP}{2TP+FP+FN}$$ (22)

$$Sensitivity=\frac{TP}{TP+FN}$$ (23)

Where TP refers to true positives, FP represents false positives, TN denotes true negatives, FN refers to false negatives, and m stands for the total number of different classes.

### 3.3 Experimental results

In this study, MDWC-Net was compared with other deep learning-based segmentation algorithms, including FCN-8S, DeeplabV3+ (Baban and Chaari, 2023), PSPNet (H. S. Zhao et al., 2017), U-Net, ResU-Net (Tang et al., 2024), Attention U-Net (Falk et al., 2019), TransU-Net (Chen et al., 2024), and PLU-Net (Song et al., 2024), through extensive experiments on the Spine Dataset. The experimental results were thoroughly analyzed, and a series of ablation experiments were conducted on the proposed blocks to validate their effectiveness.

#### 3.3.1 Experimental results on the spine dataset

The performance of MDWC-Net on the Spine Dataset is shown in Table 1. From the experimental results in Table 1, it can be observed that MDWC-Net demonstrates superior performance across multiple evaluation metrics. Specifically, the Dice and MIoU scores of MDWC-Net reach 89.86% and 90.53%, respectively. The Dice score is 7.58, 4.34, and 3.58 percentage points higher than those of FCN-8S, DeeplabV3+, and U-Net, indicating that MDWC-Net exhibits a stronger capability in distinguishing between classes. Furthermore, compared to the second-best model in Table 1, MDWC-Net also demonstrates its superiority, achieving a 1.65% and 2.71% increase in Dice and MIoU, respectively. In terms of two key metrics, GPA and Sensitivity, MDWC-Net also demonstrates remarkable performance, achieving excellent scores of 96.82% and 96.77%, respectively. Moreover, MDWC-Net achieves these results with fewer Params, lower FLOPs, and shorter Training_time. This fully demonstrates the efficiency and accuracy of MDWC-Net in segmentation tasks. The experimental data indicate that MDWC-Net not only identifies target regions more accurately in the spine segmentation task but also delineates the edges of the targets more precisely, effectively reducing instances of missed and incorrect segmentations.

**TABLE 1:** Quantitative performance of different models on spine dataset (highest score indicated in bold font).

Method	GPA/%	Dice/%	MIoU/%	Sensitivity/%	Params	FLOPs/G	Train_time (s/epoch)
FCN-8S [13]	94.51 ± 0.363	82.28 ± 0.232	81.35 ± 0.717	93.26 ± 0.662	70.31M	97.86	10.29
DeeplabV3+[44]	95.22 ± 0.451	85.52 ± 0.332	86.26 ± 0.262	94.66 ± 0.956	59.72M	175.48	13.36
PSPNet [45]	95.43 ± 0.277	85.73 ± 0.316	86.13 ± 0.365	94.21 ± 0.763	40.78M	120.06	10.88
U-Net [17]	95.82 ± 0.356	86.28 ± 0.419	86.96 ± 0.256	94.75 ± 0.462	28.95M	133.19	8.26
ResU-Net [24]	95.63 ± 0.825	86.89 ± 0.515	87.25 ± 0.523	95.02 ± 0.611	33.61M	189.81	8.85
Attention U-Net [22]	96.24 ± 0.763	87.63 ± 0.434	87.82 ± 0.305	95.31 ± 0.573	7.49M	108.85	7.74
TransU-Net [46]	96.35 ± 0.476	87.86 ± 0.543	87.56 ± 0.676	95.57 ± 0.218	46.74M	56.29	10.18
PLU-Net [47]	96.13 ± 0.203	88.21 ± 0.523	87.14 ± 0.439	95.33 ± 0.371	7.98M	120.76	9.56
MDWC-Net	**96.82** ± **0.289**	**89.86** ± **0.356**	**90.53** ± **0.315**	**96.77** ± **0.212**	**3.58M**	**23.18**	**4.82**

Note: GPA, global pixel accuracy; MIoU, mean intersection over union; Params, Parameters; FLOPs, Floating Point Operations.

**FIGURE 6:**
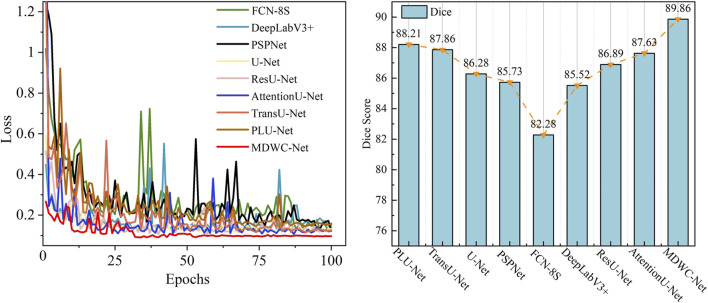
Results of different models on the spine dataset.

**FIGURE 7:**
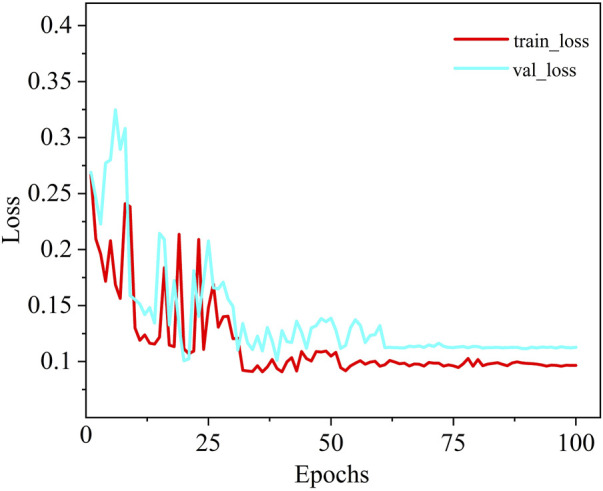
The loss curve of MDWC-Net on the spine dataset.

As shown in Figure 6, a comparison of the training loss convergence and Dice scores on the test set for each model is presented. After 100 training epochs, the MDWC-Net model demonstrates a more stable and efficient convergence speed. Moreover, as the number of iterations increases, the efficient convergence of MDWC-Net further proves that effective parameter optimization can be achieved even without pre-training. This achievement is attributed to its network design, which integrates multi-scale and information-complementary feature representations. Figure 7 shows the detailed training and validation curves of MDWC-Net. The training loss decreases steadily from approximately 0.2667 to 0.096, while the validation loss follows a similar trend, stabilizing around 0.112 after 61 epochs. The close alignment between training and validation curves indicates that MDWC-Net achieves good generalization without significant overfitting issues.

**FIGURE 8:**
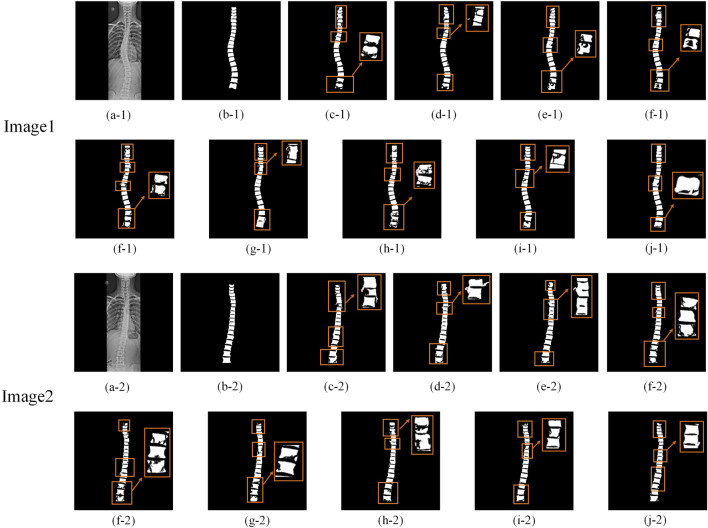
Comparison of segmentation results. The rectangular boxes highlight significant segmentation differences among models. **(a)** Original image; **(b)** Ground Truth (GT); **(c–j)** Segmentation results of FCN-8s, DeepLabV3+, PSPNet, U-Net, ResU-Net, Attention U-Net, TransU-Net, PLU-Net, and MDWC-Net, respectively, on the Spine dataset.

Additionally, to visually assess the accuracy of spine region segmentation, Figure 8 presents a comparative visualization of the segmentation results from different algorithms on the Spine Dataset. As shown in Figure 8, more accurate segmentation results are achieved by MDWC-Net. Specifically, other algorithms generally exhibit significant loss of the spine region in the segmentation output, particularly at the edges and finer structures of the spine. This issue is primarily attributed to the limitations of these algorithms in capturing spine location information beyond the receptive field and in handling finer details within the images.

In contrast, the MDWC-Net algorithm effectively alleviates this problem by integrating complementary high-level semantic features and low-level texture features, resulting in more refined and accurate spine region segmentation. In Figure 8, compared to MDWC-Net, other algorithms demonstrate more pronounced over-segmentation and under-segmentation issues during the segmentation process. Over-segmentation occurs when unnecessary details are incorrectly labeled as part of the spine region, while under-segmentation leads to the omission of key spinal structures. The network’s ability to focus on relevant features and utilize global information is enhanced by MDWC-Net through the innovative introduction of a multi-scale adaptive weighting block, a dual-feature complementary block, and a bottleneck information enhancement block. As a result, MDWC-Net outperforms other algorithms in terms of both segmentation accuracy and completeness, demonstrating exceptional performance in the spine X-ray image segmentation task.

#### 3.3.2 Significance testing of segmentation performance

To verify whether the performance improvement of the proposed MDWC-Net over other models (such as DeeplabV3+, PSPNet, U-Net, Attention U-Net, TransU-Net, and PLU-Net) is statistically significant, we conducted paired t-tests on the Dice and MIoU metrics across these models on the Spine dataset. The significance level was set to α = 0.05. As shown in Table 2, all p-values are much smaller than 0.01, indicating that the improvements of MDWC-Net in both Dice and MIoU are statistically significant. These results further demonstrate the effectiveness and robustness of the proposed method.

**TABLE 2:** Paired t-test results between MDWC-Net and other models.

Compared model	Dataset	Metric	P-value	Significant (α = 0.05)
Deeplabv3+	Spine dataset	MIoU	0.006	Yes
PSPNet	Spine dataset	MIoU	0.003	Yes
U-Net	Spine dataset	Dice	0.007	Yes
Attention U-Net	Spine dataset	MIoU	0.005	Yes
TransU-Net	Spine dataset	Dice	0.008	Yes
PLU-Net	Spine dataset	Dice	0.004	Yes

#### 3.3.3 Ablation experiment

To further explore the contribution of each block in this study, we conducted a series of ablation experiments using the Spine Dataset. First, the U-Net model was used as the baseline. Then, the multi-scale convolution block with the adaptive weighting mechanism removed was integrated into the baseline, referred to as “+Multi-scale Conv only.” The complete Multi-scale Convolution Adaptive Weighting (MSCAW) block was then integrated into the baseline, named “+MSCAW only.” Next, to verify the effectiveness of different branches in the Bottleneck Information Enhancement Block (BIEB), we tested configurations with “+MSCAW+BIEB (upper)” using only the upper branch of BIEB, and “+MSCAW+BIEB (lower)” using only the lower branch. The “+MSCAW+BIEB (full)” configuration integrates both the MSCAW block and the complete BIEB. Finally, “+MSCAW+DFCB” combines the MSCAW block with the Dual Feature Complementary Block. In addition, to further validate the effectiveness and advantages of the proposed modules, we designed replacement experiments: “w/ASPP” replaces the MSCAW module with the classical ASPP module, and “w/CBAM” replaces the BIEB module with the CBAM module. The relevant experimental results are shown in Table 3.

**TABLE 3:** Ablation experiments of each component block (highest score indicated in bold font).

Methods	MSCAW	BIEB	DFCB	Spine dataset			
				GPA/%	Dice/%	MIoU/%	Sensitivity/%
Baseline (U-Net)	✕	✕	✕	95.82 ± 0.156	86.28 ± 0.219	86.96 ± 0.256	94.75 ± 0.162
+Multi-scale Conv only	✓	✕	✕	96.21 ± 0.265	87.81 ± 0.163	87.69 ± 0.374	95.02 ± 0.149
+MSCAW only	✓	✕	✕	96.46 ± 0.117	88.65 ± 0.124	88.28 ± 0.097	95.43 ± 0.218
+MSCAW+BIEB (upper)	✓	✓	✕	96.63 ± 0.156	89.03 ± 0.091	88.73 ± 0.182	95.86 ± 0.145
MSCAW+BIEB (lower)	✓	✓	✕	96.57 ± 0.036	88.97 ± 0.083	89.16 ± 0.045	95.79 ± 0.095
+MSCAW+BIEB (full)	✓	✓	✕	96.71 ± 0.063	89.43 ± 0.041	89.56 ± 0.109	96.28 ± 0.197
+MSCAW+DFCB	✓	✕	✓	96.65 ± 0.153	89.55 ± 0.057	89.68 ± 0.036	96.31 ± 0.103
MDWC-Net	✓	✓	✓	96.82 ± 0.289	89.86 ± 0.356	90.53 ± 0.315	96.77 ± 0.212
w/ASPP	✕	✓	✓	95.96 ± 0.126	88.97 ± 0.415	89.22 ± 0.312	95.12 ± 0.217
w/CBAM	✓	✕	✓	96.27 ± 0.332	89.35 ± 0.253	89.85 ± 0.221	96.02 ± 0.366

Note: MSCAW, Multi-scale Convolution Adaptive Weighting; BIEB, bottleneck information enhancement block; DFCB, dual feature complementary block; ASPP, atrous spatial pyramid pooling; CBAM, convolutional block attention module.

From the experimental results in Table 3, it can be observed that after integrating the multi-scale convolution adaptive weighting block, the model’s performance was significantly improved, with Dice and MIoU rising to 88.65% and 88.28%, respectively. This improvement is attributed to the key role of this block in feature weighting and decision-making. The block fuses features from different scales and dynamically adjusts the scale weights of each channel based on the regional characteristics of the input image, enabling the model to more accurately capture multi-scale detail information. In addition, the combination of different branches of the Bottleneck Information Enhancement Block with the multi-scale convolution adaptive weighting block led to improvements in the network’s performance across all evaluation metrics. This enhancement is attributed to the bottleneck block’s ability to extract and utilize global contextual feature information.

Furthermore, the integration of the Dual Feature Complementary Block also contributed to the improvement in network performance. Specifically, the embedding of the DFCB resulted in the model’s GPA, Dice, MIoU, and Sensitivity increasing to 96.65%, 89.55%, 89.68%, and 96.31%, respectively. The results show that this block effectively utilizes high-level features to guide low-level features in selecting key information, reducing the loss of important information and interference from irrelevant data. And the proposed modules also outperformed classical counterparts in segmentation performance, further supporting their design rationality and task-specific effectiveness. Finally, through the integration of all blocks, MDWC-Net achieved optimal segmentation performance, and the experimental results strongly validate the effectiveness and practicality of the designed blocks.

## 4 Discussion

To further evaluate the generalization capability of the proposed MDWC-Net beyond spinal X-ray segmentation, we conducted additional experiments on two publicly available datasets: Chest X-ray dataset for lung field segmentation and ISIC2016 dataset for skin lesion segmentation. These datasets represent two distinct directions of generalization: Chest X-rays are anatomically and radiologically similar to spinal X-rays, while ISIC2016 features highly heterogeneous textures and modalities.

### 4.1 Experimental results on the chest X-ray dataset

The Chest X-ray dataset provides pixel-level annotations of lung fields. Chest radiographs share similar grayscale distribution and imaging characteristics with spinal X-rays. As shown in Table 4, it can be observed that MDWC-Net achieved a Dice coefficient of 85.32% and MIoU of 86.09%, surpassing baseline methods including U-Net and DeepLabV3+. These results demonstrate that the proposed architecture generalizes effectively not only within the spinal domain but also to other thoracic structures captured by similar imaging modalities. Such findings highlight the potential of MDWC-Net for broader applications in skeletal and soft-tissue segmentation tasks within the domain of radiography.

**TABLE 4:** Quantitative performance of each model on the chest X-ray dataset (highest score indicated in bold font, GPA represents Global Pixel Accuracy).

Method	GPA/%	Dice/%	MIoU/%	Sensitivity/%	Params	FLOPs/G	Train_time (s/epoch)
FCN-8S [13]	87.51 ± 0.963	79.22 ± 0.623	77.56 ± 0.434	88.28 ± 0.391	70.31M	97.86	22.17
DeeplabV3+[44]	89.26 ± 0.726	83.08 ± 0.986	82.88 ± 0.262	90.06 ± 0.586	59.72M	175.48	24.63
PSPNet [45]	88.58 ± 0.745	78.21 ± 0.652	78.65 ± 0.668	87.36 ± 0.197	40.78M	120.06	21.56
U-Net [17]	89.09 ± 0.187	80.15 ± 0.224	78.82 ± 0.128	90.17 ± 0.362	28.95M	133.19	17.33
ResU-Net [24]	89.95 ± 0.721	81.27 ± 0.366	82.02 ± 0.127	90.13 ± 0.523	33.61M	189.81	17.69
Attention U-Net [22]	90.12 ± 0.878	82.65 ± 0.772	81.72 ± 0.553	89.38 ± 0.963	7.49M	108.85	16.74
TransU-Net [46]	92.02 ± 0.721	81.92 ± 0.886	80.85 ± 0.262	90.63 ± 0.928	46.74M	56.29	20.16
PLU-Net [47]	91.93 ± 0.218	81.86 ± 0.672	80.77 ± 0.521	89.96 ± 0.631	7.98M	120.76	18.13
MDWC-Net	**92.75** ± **0.162**	**85.32** ± **0.183**	**86.09** ± **0.235**	**92.08** ± **0.126**	**3.58M**	**23.18**	**14.26**

Note: GPA, global pixel accuracy; MIoU, mean intersection over union; Params, parameters; FLOPs, floating point operations.

### 4.2 Experimental results on the ISIC2016 dataset

The ISIC2016 dataset contains various types of skin lesions, including melanoma and basal cell carcinoma, with high-quality pixel-level annotations. Unlike spinal X-rays, skin lesion images exhibit irregular shapes, blurry boundaries, and strong variability in texture and contrast, posing distinct challenges to segmentation models. Applying MDWC-Net to this domain allows us to evaluate its robustness across structurally unrelated medical tasks. As shown in Table 5, MDWC-Net achieved excellent results on the ISIC2016 dataset, with a GPA of 95.96%, Dice coefficient of 87.25%, MIoU of 86.75%, and Sensitivity of 94.61%, outperforming several state-of-the-art models—including a 4.83% and 5.28% improvement in GPA and Sensitivity over FCN-8s, and a 3.28% and 3.33% gain in Dice and MIoU over U-Net. Moreover, compared with the Transformer-based TransUNet, MDWC-Net achieved consistent improvements of 1.58%, 2.12%, and 2.69% in GPA, Dice, and MIoU, respectively.

**TABLE 5:** Quantitative performance of each model on the ISIC2016 dataset (highest score indicated in bold font, GPA represents Global Pixel Accuracy).

Method	GPA/%	Dice/%	MIoU/%	Sensitivity/%	Params	FLOPs/G	Train_time (s/epoch)
FCN-8S [13]	91.13 ± 0.213	82.32 ± 0.416	81.86 ± 0.658	89.33 ± 0.446	70.31M	97.86	28.89
DeeplabV3+[44]	92.56 ± 0.317	82.16 ± 0.596	80.37 ± 0.662	88.16 ± 0.263	59.72M	175.48	32.16
PSPNet [45]	91.78 ± 0.426	81.52 ± 0.776	80.96 ± 0.635	87.75 ± 0.747	40.78M	120.06	28.68
U-Net [17]	92.95 ± 0.367	83.97 ± 0.348	83.42 ± 0.514	91.28 ± 0.612	28.95M	133.19	23.42
ResU-Net [24]	93.76 ± 0.537	84.51 ± 0.526	84.28 ± 0.564	92.06 ± 0.346	33.61M	189.81	24.08
Attention U-Net [22]	93.42 ± 0.621	84.77 ± 0.597	82.15 ± 0.757	91.93 ± 0.808	7.49M	108.85	22.15
TransU-Net [46]	94.38 ± 0.812	85.13 ± 0.903	84.06 ± 0.795	90.27 ± 0.615	46.74M	56.29	27.53
PLU-Net [47]	94.12 ± 0.253	85.33 ± 0.434	84.88 ± 0.652	91.63 ± 0.389	7.98M	120.76	25.86
MDWC-Net	**95.96** ± **0.319**	**87.25** ± **0.463**	**86.75** ± **0.577**	**94.61** ± **0.362**	**3.58M**	**23.18**	**18.16**

Note: GPA, global pixel accuracy; MIoU, mean intersection over union; Params, parameters; FLOPs, floating point operations.

**FIGURE 9:**
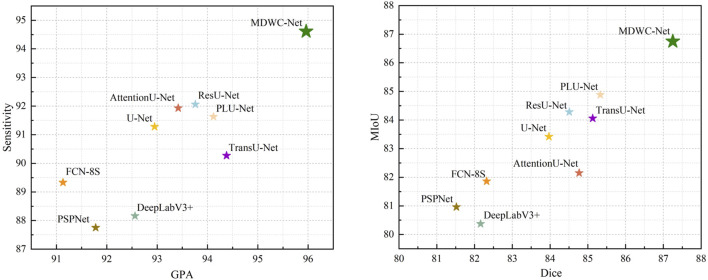
Distribution of scores for different models.

These findings demonstrate that the multi-scale feature modeling and dynamic information fusion mechanisms in MDWC-Net are effective not only for structured anatomical regions like the spine but also for unstructured lesion segmentation tasks. To better visualize model performance, Figure 9 shows that MDWC-Net consistently appears in the top-right region of the GPA–Sensitivity and Dice–MIoU scatter plots, indicating a strong trade-off between accuracy and robustness, and confirming its generalizability across diverse medical image modalities.

## 5 Conclusion

This study proposes MDWC-Net, an efficient deep network designed for spinal X-ray image segmentation. By incorporating multi-scale convolution adaptive weighting, dual feature complementary block, and bottleneck information enhancement block, the model demonstrates outstanding segmentation performance with strong generalization capability and deployment potential. Although this work primarily focuses on improving segmentation accuracy, its high-quality structural boundary extraction also provides a reliable basis for downstream clinical tasks such as spinal parameter measurement and preoperative path planning. In addition, the lightweight design and low computational cost make it suitable for integration into radiology-assisted reading systems or surgical planning platforms. While MDWC-Net demonstrates robust performance across diverse imaging conditions, further optimization could enhance its effectiveness in extremely challenging scenarios such as very low-contrast or heavily degraded X-ray images. Future work will incorporate clinical user feedback for prospective validation, optimize model deployment through techniques such as model pruning and knowledge distillation, and focus on enhancing robustness under challenging imaging conditions to meet diverse clinical requirements.

## Data Availability

The raw data supporting the conclusions of this article will be made available by the authors, without undue reservation.
